# Personalized Therapy and Clinical Outcome for Heart Failure

**DOI:** 10.3390/jcm11164851

**Published:** 2022-08-18

**Authors:** Alvaro Aceña, Javier de Juan Bagudá, Luis M. Rincón

**Affiliations:** 1 Department of Cardiology, Hospital Universitario Fundación Jiménez Díaz, 28040 Madrid, Spain; 2 Department of Medicine, Universidad Autónoma de Madrid, 28049 Madrid, Spain; 3 Department of Cardiology, Hospital Universitario 12 de Octubre, Instituto de Investigación Sanitaria Hospital 12 de Octubre (imas12), 28041 Madrid, Spain; 4 Centro de Investigación Biomédica en Red de Enfermedades Cardiovasculares (CIBERCV), 28029 Madrid, Spain; 5 Facultad de Medicina, Universidad Europea, 28670 Madrid, Spain; 6 Instituto de Investigación Biomédica de Salamanca (IBSAL), Complejo Asistencial Universitario de Salamanca, 37007 Salamanca, Spain; 7 Departamento de Medicina, Universidad de Salamanca, 37007 Salamanca, Spain

Heart failure (HF) is a complex clinical syndrome that results from the structural and/or functional impairment of systolic function or ventricular filling, which in turn causes elevated intracardiac pressure and/or inadequate cardiac output at rest and/or during exercise. It has a chronic nature, with progression characterized by the development of signs and symptoms that greatly impact quality of life and reduce life expectancy.

HF poses a major challenge to health systems in developed countries; it is the leading cause of hospitalizations in persons aged above 65, and it has an estimated worldwide prevalence of >64.3 million cases [1]. In the USA, there is an annual incidence of 870,000 new cases, and 6 million people live with HF (~1.8% of population) [2]. In addition, HF has significant mortality, reaching—in the last published clinical trials—rates of more than 11% in two years in the beneficial treatment arms [3] of those with reduced EF, and more than 14% in cases of preserved EF [4].

The personalization of therapy for HF patients is mainly based on left ventricular ejection fraction (EF), the etiology of HF, and patient comorbidities.Currently, the most commonly used criterion to classify HF is EF, with guidelines proposed in Europe of three categories representing phenotypes with differential clinical characteristics: HF with reduced EF (HFrEF) when EF is below 40%;HF with mildly reduced or mid-range EF for EF 40–49%;and HF with preserved EF (HFpEF) for EF ≥50%. While morbidity and survival are similarly limited across the spectrum of EF, this classification isjustified, as the clinical benefits associated with treatment havebeen classically limited to patients with HFrEF. Still, most of the drugs used in relation to HF have no or modest effects in patients with preserved or mildly reduced EF.

This reflects the need to personalize therapy based on underlying etiology. The main causes of HF are ischemic heart disease (26.5%), followed by hypertensive heart disease (26.2%), cardiomyopathy (6.5%), mitral valve disease (2.7%), alcoholic cardiomyopathy (2.4%), aortic valve disease (2.3%), and myocarditis (1.7%) [1]. All these entities should be treated differently, and although many of the treatments for heart failure are common (e.g., diuretics, vasodilators, ISGLT2, etc.), the way they are implemented, and the particularities of each etiology, make each patient a different subject that requires personalized therapy.

Biomarkers emerge as one of the major breakthroughs for personalized medicine in HF [5]. Beyond the routine use of natriuretic peptides and cardiac troponins, recent advancesin the fields ofgenomics, metabolomics, transcriptomics, and proteomicsshould be highlighted. The role of -omics extends from diagnosisfor the precise characterization of genetically driven cardiomyopathies, to prognostic purposes, where several emerging biomarkers emerge as predictors of HF events in specific settings [6]. Genetic testing and other -omics are used to select treatment strategies (such as defibrillators forHF secondary to malignant mutations), tounravel phenotype–genotype interactions (by identifying novel HF pathways or differentiating subtypes of HF), or to titrate several medications. Some of them even hold the promise of turning into novel therapeutic targets. The novel information provided by biomarkers and -omics is inherently linked to personalized and precision medicine.

Personalized therapy forHF does not extend only to pharmacological treatment, as remarkable progress has beennoted in the field of medical devices. Thus, there are wide alternatives in the stimulation section, from olddevices, such as implantable cardioverter–defibrillators or resynchronizationtherapy, to newer ones, such as left bundle branch area pacing [7], cardiac contractility modulation [8], or baroreflex activation therapy [9]. A percutaneous approach for the treatment of HF secondary to mitral or tricuspid regurgitation is increasingly performed with the advent of transcatheter edge-to-edge leaflet repair, improving prognosis in selected patients [10,11], and some patients with advanced HF benefit from left ventricular assist devices [12]. In thismodern era, devices can also be useful tools in the follow-up of patients, for example, to identify those with impending HF decompensation, in order to avoidhospitalizations [13,14].

In addition, associated with the main causes of HF, we find two fundamental pillars when adjusting the different treatments available to our patients. On the one hand, there are patients’ comorbidities, and on the other hand, the volemia situation with which we find the patients.

Regarding patients’ comorbidities, we must consider situations such as: (1) obesity (difficulty in physical examination, worse ultrasoundwindow, lower natriuretic peptides, etc.),which obstructs reaching an euvolemic status, and therefore prevents the identification of the best functional class; (2) chronic renal disease, given that in advanced situations the use of therapies that modify the prognosis of HF may be limited or contraindicated, sometimes requiring the use of less robust hydralazine ornitrates instead of ACEi/ARB/ARNi, and sometimes MRAs or SGLT2i cannot be used; (3) asthma, the presence of which is a relative contraindication for the use of betablockers (BBs) and low doses of cardioselective BBs should be used [15].

When evaluating patients’ blood volume, it should be taken into account that in patients with dyspnea, at rest, the patient must be “dried out” to bring themto euvolemia and study the etiology of this circumstance, and once these two problems have been solved, therapy can be started tomodify the prognosis of HF. It is very important to understandthat if we use BBs in this acute phase before time, the patient may decompensate again, so the time to start BBs, if indicated, after hospital admission for the decompensation of HF, should be perfectly monitored clinically to avoid unnecessary problems.

For all of these reasons, it is essential to individualize the therapeutic strategies for each patient diagnosed with HF. Thepresent Special Issue aims to contribute to the management of this complex syndrome.
